# Supplementary material for: Active learning potentials for first-principles phase diagrams using replica-exchange nested sampling
Source: NPJ Comput Mater. 2026 Feb 5;12(1):107. doi: 10.1038/s41524-026-01989-z (PMC12982124; doi:10.1038/s41524-026-01989-z)
Supplement: Supplementary file 1 — Supplementary information [file 41524_2026_1989_MOESM1_ESM.pdf]

Active learning potentials for first-principles phase diagrams using replica-exchange nested sampling

Supplementary Material

(Dated: January 7, 2026)

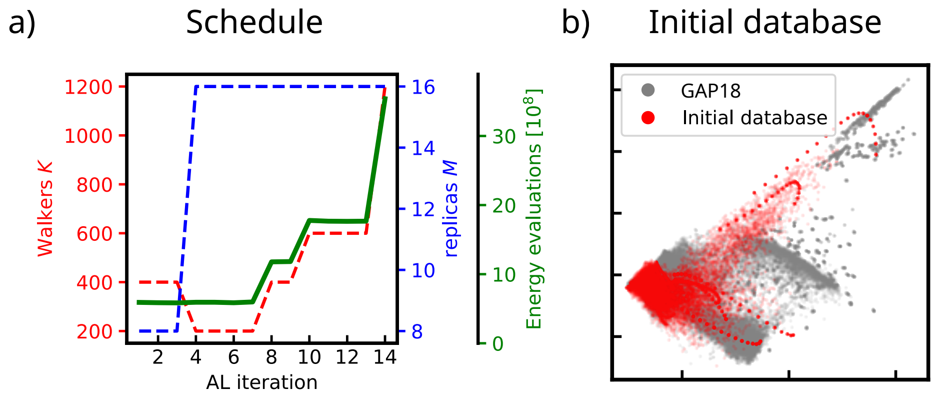

FIG. S1. RENS hyperparameter schedule and principal component analysis (PCA) of the atomic environments contained in the initial database for Ge.

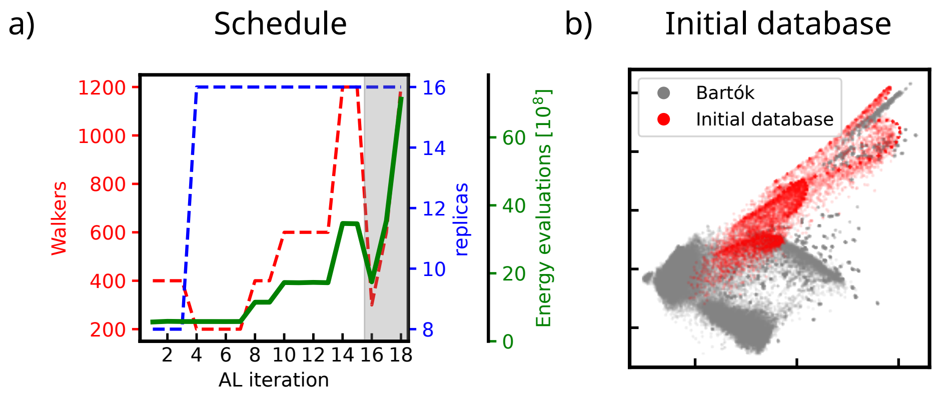

FIG. S2. RENS hyperparameter schedule and principal component analysis (PCA) of the atomic environments contained in the initial database for Ti.

SCHEDULES AND INITIAL DATABASE ENVIRONMENTS FOR GE AND TI

The parameter schedules for the germanium and titanium active learning (AL) strategies are shown in Figs. S1 and S2.

ESTIMATED GPU AND CPU TIMES OF THE AL PROCEDURE

To provide an impression of the computational resources required for the numerical experiments presented in this work, Fig. S3 shows a timeline of the silicon active-learning (AL) run. The reported timings include queueing times, which were negligible compared to the overall wall-clock time.

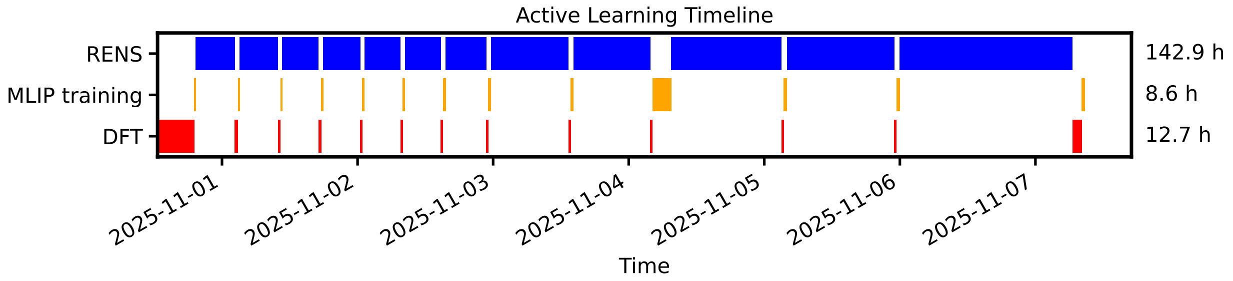

FIG. S3. Timeline of the silicon AL run.

The density-functional theory (DFT) calculations were executed on different compute nodes equipped with processors of comparable performance to an Intel(R) Xeon(R) Gold 6230 CPU @ 2.10 GHz. At any given time, the number of concurrent DFT calculations was limited such that at most 200 CPU cores were used in parallel. The total elapsed wall-clock time of the DFT simulations amounts to approximately 266.6 h. With 8 CPU cores employed per calculation, this corresponds to a total CPU consumption of about 2133 CPU h, consistent with the 12.7 h shown in Fig. S3 multiplied by the maximum parallel core count.

The MLIP training constitutes the least expensive component of the workflow, requiring a total of 8.6 h on a single NVIDIA A40 GPU. By far the most computationally demanding part of the AL procedure is the RENS sampling. These simulations were carried out on an NVIDIA A40 node using four GPUs in parallel, resulting in a total GPU usage of 571.6 GPU h for the RENS component.

SYSTEM SIZE AND THE RENS OVERLAP PROBLEM

In the main manuscript, we applied Replica-exchange nested sampling (RENS) successfully to systems containing up to 32 atoms. Scaling the system sizes, however, is in general a challenging problem in the framework of RENS. In the following, we discuss the computational cost of RENS simulations and the resulting implications for scaling.

Increasing the system size naturally increases the computational cost of NS. Both the number of iterations—and hence the number of configurations that must be sampled—as well as the cost of each energy evaluation scale linearly with system size. The overall computational cost of NS therefore grows roughly as $\mathcal{O}(N_{\text{atoms}}^2)$ [1].

With RENS an additional difficulty arises: phase transitions become increasingly sharp with

growing system size, making it substantially harder to maintain sufficient overlap between replicas. It is generally found that keeping the swap acceptance finite in replica-exchange schemes requires the spacing of adjacent control-parameter values to decrease with N_{atoms}. In parallel tempering, this leads to $\Delta\beta \propto 1/\sqrt{N}$ under regularity conditions such as a locally constant heat capacity, and thus to an overall $\mathcal{O}(\sqrt{N})$ scaling of the number of replicas [2, 3]. However, near phase transitions the heat capacity varies sharply, and this approximation breaks down: much denser spacing is required in the vicinity of phase transitions to preserve overlap between neighboring replica distributions.

In RENS, the situation is even more intricate. Replica exchanges couple likelihood-constrained prior distributions that evolve at every iteration, precluding a simple or static scaling law. Since RENS is explicitly designed to traverse phase boundaries, the adaptive tuning of control-parameter (e.g. pressure) spacings remains a central computational challenge, and becomes more severe as the system size increases.

In Fig. S4 we demonstrate manifestations of the overlap problem in practice, how they can be detected using the swap acceptance rates as heuristics and how they can be systematically overcome. In Fig. S4a, using $M = 8$ replicas between 2 and 16 GPa, overlap is lost between replicas 3 and 4 during the first half of the simulation, leading to biased sampling and discontinuities in the melting line as seen in C_P and $\overline{Q_4}$. When trying to circumvent this shortcoming by decreasing the pressure spacing in the pressure range of 8–19 GPa, a similar behavior is observed as depicted in Fig. S4b. Sampling at irregular intervals focused on the transition region still fails to maintain overlap between replicas 0 and 1. Additionally, a large discrepancy in the melting temperatures at 8 GPa between Figs. S4a and b can be observed. Together these observations highlight two key insights: sufficient overlap is (i) required near specific pressures for the liquid to transition into the correct solid phase, and (ii) critical for the quantitative prediction of transition temperatures. Using a significantly denser and regular pressure grid between 1 and 16.5 GPa (see Fig. S4c) restores overlap across the liquid–solid transition and yields a consistent melting line. Nevertheless, overlap is again lost between replicas 17 and 18, leading to biased sampling across the high-pressure solid–solid transition. The resulting discontinuity in $\overline{Q_4}$ appears as an artificial vertical boundary between the cubic diamond ($Fd\bar{3}m$) and simple hexagonal ($P6/mmm$) phases. This effect is more severe than for the liquid–solid transition due to the large structural and energetic contrast between the two phases.

The observations above, arising from the loss of overlap in RENS, ultimately stem from biased sampling of the likelihood-constrained prior distributions, analogous to the failures observed in

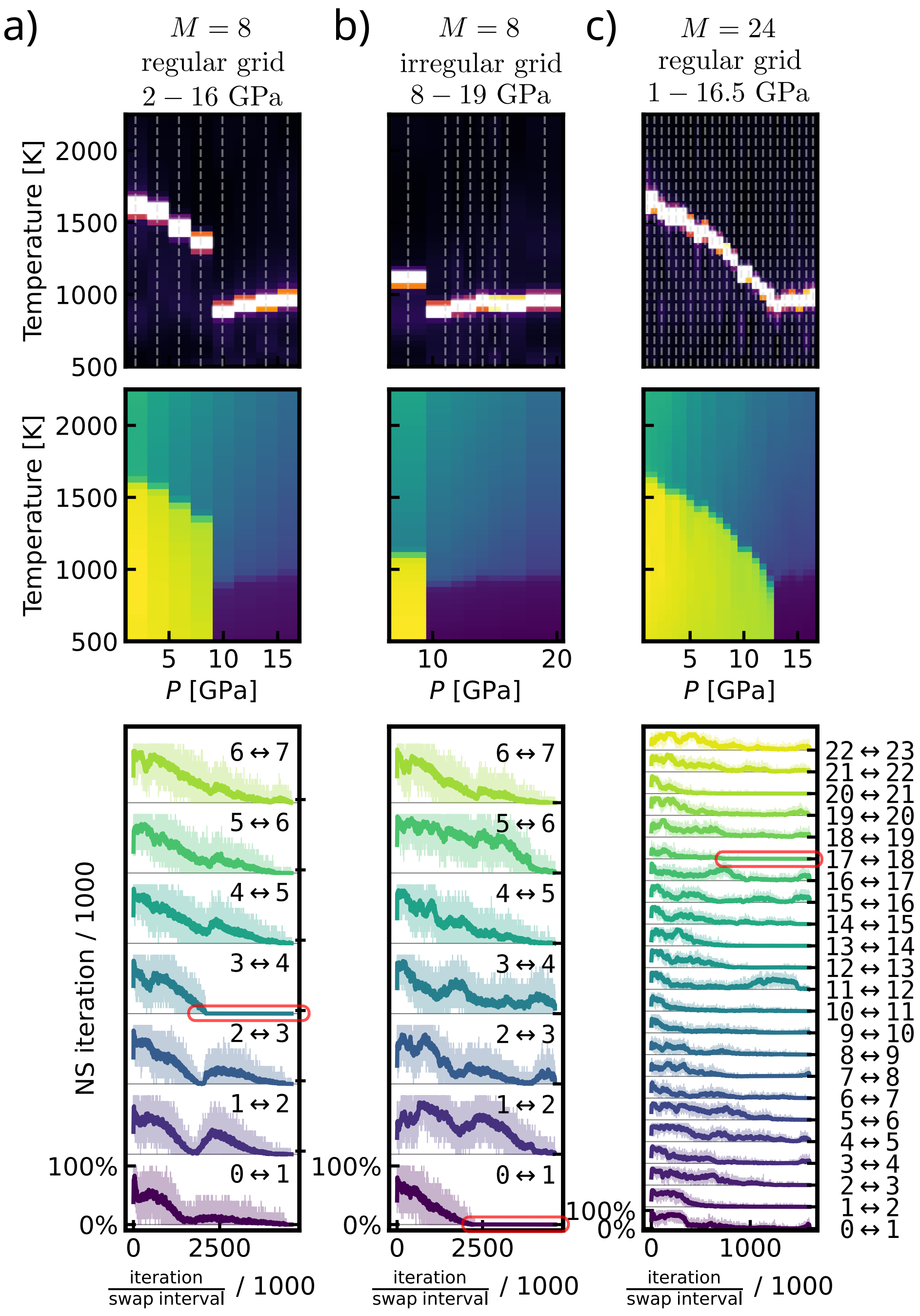

FIG. S4. Demonstration of the failure of RENS due to loss of overlap between replicas. Top panels show the constant pressure heat capacity C_P, middle panels show the $\overline{Q_4}$ order parameter and bottom panels show the RENS *inter*-swap acceptance rates. We compare three different $N_{\mathrm{atoms}} = 32$ Si runs with different choices of the number of replicas M and the way the pressure intervals are chosen. a) $K = 600$, $M = 8$ with regular grid between 2 and 16 GPa. b) $K = 600$, $M = 8$ with irregular grid between 8 and 20 GPa focussed around the transition region. c) $K = 200$, $M = 24$ with regular grid between 1 and 16.5 GPa. Red boxes indicate vanishing acceptance rates indicating a loss of overlap.

independent NS simulations [4]. This issue is particularly severe for materials such as silicon or germanium, whose phase transitions are extremely sharp. In such systems, the RENS overlap problem poses a central obstacle to extending AL strategies to larger system sizes.

In the main manuscript we demonstrated that a loss of overlap can, to some extent, be systematically mitigated by reducing the external-parameter spacings in critical regions. For silicon, where we discussed several artifacts arising from overlap loss in Fig. S4, it is sufficient to maintain pressure spacings of 2 GPa at low pressures, while substantially refining the grid to 0.25 GPa in the vicinity of the transition. This refinement restores persistent overlap between replicas and thereby recovers correct sampling behavior.

[1] L. B. Pártay, G. Csányi, and N. Bernstein, Nested sampling for materials, Eur. Phys. J. B **94**, 159 (2021).

[2] D. A. Kofke, On the acceptance probability of replica-exchange Monte Carlo trials, The Journal of Chemical Physics **117**, 6911 (2002).

[3] A. Kone and D. A. Kofke, Selection of temperature intervals for parallel-tempering simulations, The Journal of Chemical Physics **122**, 206101 (2005).

[4] N. Unglert, L. B. Pártay, and G. K. H. Madsen, Replica Exchange Nested Sampling, J. Chem. Theory Comput. 10.1021/acs.jctc.5c00588 (2025).
